# Optimizing Taxonomic Assignments and Community State Types for the Vaginal Microbiota in Older Women

**DOI:** 10.1093/geroni/igaf122.4408

**Published:** 2025-12-31

**Authors:** Sarah Brown, Michael France, L Elaine Waetjen, Michelle Shardell, Rebecca Brotman, Jacques Ravel, Johanna Holm

**Affiliations:** University of Maryland School of Medicine, Baltimore, Maryland, United States; University of Maryland School of Medicine, Baltimore, Maryland, United States; University of California Davis Health, Sacramento, California, United States; University of Maryland School of Medicine, Baltimore, Maryland, United States; University of Maryland School of Medicine, Baltimore, Maryland, United States; University of Maryland School of Medicine, Baltimore, Maryland, United States; University of Maryland School of Medicine, Baltimore, Maryland, United States

## Abstract

The vaginal microbiome plays a critical role in protecting urogenital health across the lifespan, yet bioinformatic tools and community state type (CST) definitions derived from 16S rRNA gene amplicon sequencing data have been developed using data from reproductive-age women. As a result, current classification tools may not accurately describe the microbial compositions of peri- and postmenopausal women, whose vaginal microbiota shift significantly with declining estrogen levels. To address this gap, we refined two previously published key tools, vSpeciateDB and VALENCIA, using sequencing data from N = 1924 peri/postmenopausal women aged 45-72 who were enrolled in the Study of Women Across the Nation (SWAN) and the Human Papillomavirus in Perimenopause (HIP) study. We expanded the three region-specific vSpeciateDB databases with 3,092 high-confidence sequences, adding 826, 710, and 572 new species to the V1V3, V3V4, and V4 databases, respectively. This improved species-level classification rates for older women from 95% to 98%. Hierarchical clustering of 2281 peri/postmenopausal samples revealed three novel sub-CSTs of a CST previously described in reproductive-age women as low in Lactobacillus spp, moderate Prevotella abundance and high evenness of other species. These sub-CSTs differed in relative abundances of key taxa, including Prevotella, Finegoldia, Corynebacterium, Faecalibacterium, Fenollaria, and Phocaeicola, and accounted for 24.3% of peri/postmenopausal samples, compared to 1.4% in reproductive-aged women. Including these sub-CSTs improved CST assignment quality for both reproductive-age and peri/postmenopausal women. Ongoing analyses will explore whether these sub-CSTs are differentially associated with health outcomes in older women including genitourinary syndrome of menopause and recurrent urinary tract infections.

